# Canine hookworm infections in Vietnam: prevalence, risk factors, and detection of zoonotic *Ancylostoma ceylanicum*

**DOI:** 10.3389/fcimb.2026.1926338

**Published:** 2026-10-28

**Authors:** Minh-Trang Thi Hoang, Dinh Ng-Nguyen, Ketsarin Kamyingkird, Van-Phuong Ngo, Tawin Inpankaew

**Affiliations:** 1 Department of Parasitology, Faculty of Veterinary Medicine, Kasetsart University, Bangkok, Thailand; 2 Department of Microbiology and Parasitology, Buon Ma Thuot Medical University, Buon Ma Thuot, Dak Lak, Vietnam; 3 Department of Veterinary Medicine, Faculty of Agriculture, Tay Nguyen University, Buon Ma Thuot, Dak Lak, Vietnam; 4 Department of Basic Sciences, Buon Ma Thuot Medical University, Buon Ma Thuot, Dak Lak, Vietnam

**Keywords:** *Ancylostoma*, canine hookworms, dogs, molecular identification, Vietnam

## Abstract

**Introduction:**

Canine hookworms are widely distributed globally and represent a public health concern because of their zoonotic potential. Hookworm infection is endemic in Vietnam; however, information on canine hookworm infections remains limited. This study investigated the prevalence, associated factors, species identification of canine hookworms, and genetic diversity of *Ancylostoma ceylanicum* in dogs from a rural area of Vietnam.

**Methods:**

A total of 371 fecal samples were collected from dogs and examined using fecal flotation with Sheather’s solution. Hookworm positive samples were further subjected to polymerase chain reaction (PCR) for species identification.

**Results:**

Hookworm infection was detected in 66.6% of dogs (95% CI: 61.5-71.3). Dogs without a history of deworming (adjusted OR [aOR] = 1.77, 95% CI: 1.08-2.89) and lack of proper fecal management (aOR = 1.79, 95% CI: 1.01-3.17) had significantly higher odds of hookworm infection. Molecular analysis identified two hookworm species, *A. ceylanicum* and *Ancylostoma caninum*, with *A. ceylanicum* being predominant. Phylogenetic analysis based on *cox1* sequences showed that all *A. ceylanicum* sequences belonged to the zoonotic haplotype and were closely related to isolates from humans, dogs, and cats across Asia.

**Discussion:**

These findings contribute to the growing evidence of zoonotic *A. ceylanicum* circulation in dogs across Asia and support the need for integrated One Health surveillance and control measures in endemic regions.

## 1 Introduction

Canine hookworms are widespread and represent an important veterinary and zoonotic concern, particularly in tropical and subtropical regions (Kladkempetch et al., 2020). Dog hookworms include *Ancylostoma caninum*, *Ancylostoma ceylanicum*, and *Ancylostoma braziliensis*, which are commonly distributed in tropical regions, while *Uncinaria stenocephala* occurs mainly in cooler climates (Traub, 2013; Traub et al., 2004). Hookworm infection in dogs is associated with gastrointestinal disturbances and hematological abnormalities, including diarrhea, intestinal blood loss, and anemia, with severe infections potentially leading to mortality in young animals (Tenorio et al., 2024; Traversa, 2012). Aside from affecting dog health, all canine hookworms present important zoonotic risks. Humans can become infected when infective larvae penetrate the skin from contaminated soil or through exposure to environments contaminated with dog feces, including water, food, or vegetables (Tenorio et al., 2024). Several canine hookworm species are associated with different clinical manifestations in humans. *A. caninum* causes eosinophilic enteritis and cutaneous larva migrans (CLM); *A. braziliense* is the main cause of intense itching and prolonged creeping eruption due to CLM; and *U. stenocephala* causes rare CLM and mild gastrointestinal symptoms (Tahar et al., 2025). Notably, *A. ceylanicum* is the only canine hookworm species that can establish natural patent infection and eosinophilic enteritis in humans (Haydon and Bearup, 1963; Traub et al., 2021). In the Asia-Pacific region, *A. ceylanicum* is reported as the second most common hookworm infecting humans after *N. americanus* (Traub et al., 2021).

Microscopic examination of hookworm eggs and adult worms has traditionally been used for hookworm diagnosis due to its simplicity and low cost. However, morphological similarity and overlapping egg sizes among hookworm species limit reliable species identification (Singh et al., 2022; Soulsby, 1982). In addition, although adult hookworms can be differentiated morphologically, the process is time-consuming and requires personal skill (Traub et al., 2004). PCR-based diagnostics have been applied for species-level identification directly from the eggs in fecal samples (Sayasone et al., 2026; Traub et al., 2008).

Vietnam is endemic for hookworm infections, particularly in rural areas where free-roaming dogs are common, and contact between humans and dogs is frequent. Previous epidemiological surveys in Vietnam have consistently identified hookworms as one of the predominant intestinal parasites detected in dogs (Hieu et al., 2016; Na et al., 2025). In the country, most previous studies have relied on copromicroscopic examination, limiting accurate species identification and its public health relevance. A few investigations have reported on the canine hookworm species in dogs based on molecular analysis (Chuc et al., 2016; Ng-Nguyen et al., 2015; Nguyen et al., 2022), but such data remain scarce. Therefore, the present study aimed to determine the prevalence, associated factors, and species composition of canine hookworms in dogs in a rural area of Vietnam using combined copromicroscopic and molecular approaches. In addition, the genetic diversity of *A. ceylanicum* was analyzed.

## 2 Materials and methods

### 2.1 Study site and sample collection

A cross-sectional study was conducted from February to March 2025. The study was undertaken in Eatam and Eaho communes, Krong Nang district, Dak Lak province, Vietnam. A sample size was calculated using a single proportion population formula $n=\left[Z_{1-\alpha 2}^{2}p\left(1-p\right)\right]/d^{2}$, where *n* is the required sample size, *p* is the estimated prevalence of hookworm infection in dogs (40%) (Dinh and Kieu, 2022), *d* is the margin of error (0.05), and $Z_{1-\alpha 2}^{2}$ is the standard deviation at a confidence level of 95% (1.96). The calculated minimum sample size was 369; however, 371 samples were collected to ensure the minimum sample size was achieved.

In each selected commune, four hamlets were selected for sampling. Within each selected hamlet, households owning dogs were identified from a household list provided by the local authority and randomly selected for inclusion in the study. Dogs from these households were enrolled with the owner’s consent, regardless of age, sex, or breed. In households with a single dog, the dog was included in the study. In households with multiple dogs, each dog was assigned a unique identification number, and up to two dogs were randomly selected. Approximately 5–10 g of fresh feces were collected from each selected dog and immediately homogenized in 5% *w/v* potassium dichromate. The preserved samples were stored in a cool box during field collection and transportation and subsequently at 4 °C until laboratory analysis. Information on dog demographics (age, sex, and breed) and management practices was collected through interviews with dog owners using a structured questionnaire.

Animal ethics approval was obtained from The Kasetsart University Institutional Animal Care and Use Committee under the Ethical Review Board of the Office of National Research Council of Thailand (ACKU67-VET-098). In addition, informed consent was obtained from each dog owner prior to the collection of samples and data.

### 2.2 Copromicroscopic examination of hookworm eggs

Fecal samples were tested for the presence of hookworm eggs by a fecal flotation technique with Sheather’s solution (specific gravity of 1.2 g/cm^3^) as the flotation solution (Inpankaew et al., 2014a). Sheather’s sugar flotation solution was prepared fresh on the day of examination by dissolving approximately 400 g of granulated sugar in warm water, bringing the final volume to 1 L, and then cooling to room temperature. The specific gravity was adjusted to 1.2 before use. For each sample, 2 g of feces was thoroughly mixed with distilled water at a 1:4 ratio. The mixture was then strained and transferred into a 10 mL centrifuge tube. After centrifugation at 3000× g for 2 min, the supernatant was removed, and the sediment was resuspended in Sheather’s solution. Sheather’s solution was then added to the tube until a positive meniscus formed, and a coverslip was placed on top. After 10 min, the coverslip was carefully transferred to a microscope slide and examined under a light microscope at 10× and 40× magnification. The entire coverslip area was screened systematically using a zig-zag pattern to detect hookworm eggs.

### 2.3 DNA extraction

Dog fecal samples microscopically positive for hookworm eggs were subjected to DNA extraction using an E.Z.N.A.^®^ Stool DNA kit (Omega Bio-tek Inc.; Norcross, GA, USA) according to the manufacturer’s instructions, with a slight modification: the cell lysis step was performed in tubes containing beads supplied with the DNA extraction kit using a Buller Blender Lite machine (Next Advance, Inc., Troy, NY, USA) at speed 4 for 10 min to enhance the disruption of parasite eggs. Final elution of DNA was stored at -20 °C ready for molecular analysis.

### 2.4 PCR-restriction fragment length polymorphism of hookworm species

A PCR-RFLP characterization of canine hookworms was carried out using previously published primers, PCR reaction, and cycling conditions (Inpankaew et al., 2014b; Traub et al., 2008). RTGHF1 (5’-CGTGCTAGTCTTCAGGACTTTG-3’) and RTABCR1 (5’-CGGGAATTGCTATAAGCAAG TGC-3’) primers were used to amplify a 545-bp region of the internal transcribed spacer (ITS)-1, 5.8S, and ITS-2 of *A. caninum*, *A. ceylanicum*, and *U. stenocephala*. For conventional PCR, a 673-bp region of an *A. braziliense* was amplified by using RTGHF1 and a specific reverse primer, RTAYR1 (5’-CTGCTGAAAAGTCCTCAAGTCC-3’).

Each PCR reaction consisted of 4 μL of PCR Master Mix (HOT FIREPol^®^ Blend Master Mix, Solis BioDyne, Tartu, Estonia), 1 μL (10 pmol) of each primer, 2 μL of DNA template, and 12 μL of double-distilled water in a 20-μL reaction. The cycling conditions consisted of an initial denaturation at 95 °C for 15 min, 64 °C for 1 min, 72 °C for 30 s, followed by 50 amplification cycles of a denaturation step at 94 °C for 30 s, annealing at 64 °C for 30 s, and extension at 72 °C for 30 s, and a final extension at 72 °C for 7 min. Previously confirmed DNA samples for each target species were used as positive controls, while no-template controls were included as negative controls in each PCR run.

Amplified PCR products of RTGHF1-RTABCR1 were digested with *HinF*I and *Rsa*I endonucleases in separate reactions at 37 °C overnight to differentiate *A. caninum*, *A. ceylanicum*, and *U. stenocephala*. The RFLP patterns generated by each sample were then compared to the expected RFLP profiles for each hookworm species (Palmer et al., 2007).

### 2.5 PCR of the cytochrome oxidase-1 gene of *A. ceylanicum*

Positive samples for *A. ceylanicum* on the PCR-RFLP were further characterized to a haplotype level by analysis of the *cox1* gene according to (Inpankaew et al., 2014b). Primers AceyCOX1F (5′-GCTTTTGGTATTGTA-AGACAG-3′) and AceyCOX1R (5′- CTAACAACATAATAAG-TATCATG-3′) were utilized to amplify a 377 bp region of the *cox1* gene of *A. ceylanicum*.

The PCR was carried out in a 20-μL reaction, with each reaction containing 4 μL of PCR Master Mix (HOT FIREPol^®^ Blend Master Mix, Solis BioDyne, Tartu, Estonia), 1 μL (10 pmol) of each primer, 2 μL of DNA template, and 12 μL of double-distilled water. The cycling conditions consisted of an initial denaturation at 95 °C for 15 min, followed by 50 amplification cycles of a denaturation step at 94 °C for 30 s, annealing at 64 °C for 30 s, extension at 72 °C for 30 s, and a final extension at 72 °C for 7 min. Each PCR assay included confirmed *A. ceylanicum* DNA as a positive control and no-template reactions as negative controls to monitor for contamination.

### 2.6 DNA sequencing and phylogenetic analysis of *cox1* gene sequences

A total of 15 *A. ceylanicum cox1* PCR-positive samples were randomly selected and purified using the E.Z.N.A.^®^ Gel Extraction Kit (Omega Bio-tek Inc.; Norcross, GA, USA) according to the manufacturer’s instructions. Sanger sequencing was performed by U2Bio (Seoul, South Korea) with the forward primer. The resulting nucleotide sequences from a 377-base pair fragment of the *cox1* gene were read with Finch TV version 1.4.0 trace viewer (Geospiza, Inc., Seattle, WA, USA). Sequence alignment was performed in BioEdit program (version 7.7) against reference *cox1* sequences of *A. ceylanicum* isolates from Vietnam (GenBank accession no. MW549493, MW549496, MW549497, and MW549609), Malaysia (GenBank accession no. KC247734, KC247738, and PP796587), Cambodia (GenBank accession no. KF896596, KF896598 and PP350012), Thailand (GenBank accession no. LC533327, OR342784, ON430680, and ON430682), and China (GenBank accession no. KP072069, KP072073, and NC035142). *A. caninum* (GenBank accession no. NC012309) and *A. duodenale* (GenBank accession no. NC003415) were used as the outgroups. Phylogenetic analyses were then constructed using the Neighbor-joining method based on Tamura-Nei parameter distance estimates, calculated from a 317 base pair region of the aligned sequences. Bootstrap analyses were conducted using 1,000 replicates, and the phylogenetic tree was constructed using Mega 12 software (www.megasoftware.net).

### 2.7 Statistical analysis

All statistical analyses were performed using R software version 4.4.3 (R Core Team, 2025). Percentages and their corresponding 95% confidence intervals (CI) were calculated for categorical variables. Univariate associations between each explanatory variable and the outcome were first assessed using Chi-square tests. Variables with *p*-values< 0.20 in the univariate analyses were selected for inclusion in the multivariable logistic regression model. Model refinement was performed using backward elimination, with statistical significance set at *p* < 0.05. Variables excluded during the initial screening were subsequently re-evaluated and retained if their inclusion altered any regression coefficient estimates by more than 20%. Biologically plausible two-way interactions were also tested, but none reached statistical significance at the 0.05 level. Multicollinearity among independent variables was evaluated using the Variance Inflation Factor (VIF), and influential observations were identified using Cook’s distance. The goodness-of-fit of the final multivariable logistic regression model was evaluated using the Pearson chi-square test. There were no missing data among the dogs included in the analysis.

## 3 Results

### 3.1 General characteristics of the studied dogs

The study included 371 dogs, and their general characteristics are summarized in **Table 1**. Female dogs accounted for 56.3% (209/371) of the dog study population, and 59.8% (222/371) were older than 12 months. Most dogs were local breeds (88.1%) and allowed to roam freely (75.2%). In addition, 75.5% (280/371) of dogs had never been dewormed, and only a small proportion of owners reported proper management of dog feces.

**Table 1:** Univariable analysis of factors associated with hookworm infection in dogs based on copromicroscopic examination.

Variables	Number examined (%)	Number positive (%)	Crude OR (95% CI)	*P*-value
Total number of dogs	371 (100.0)	247 (66.6)		
Residence place:				
Eatam	196 (52.8)	126 (64.3)	1.00	0.322
Eaho	175 (47.2)	121 (69.1)	1.24 (0.81-1.93)	
Sex:				
Male	162 (43.7)	108 (66.7)	1.00	0.974
Female	209 (56.3)	139 (66.5)	0.99 (0.64-1.53)	
Age:				
≤ 12 months	149 (40.2)	97 (65.1)	1.00	0.621
> 12 months	222 (59.8)	150 (67.6)	1.12 (0.72-1.73)	
Breed of dog:				
Non-local	44 (11.9)	22 (50.0)	1.00	0.013
Local	327 (88.1)	225 (68.8)	2.21 (1.16-4.18)	
Confined:				
Yes	92 (24.8)	59 (64.1)	1.00	0.566
No	279 (75.2)	188 (67.4)	1.16 (0.70-1.89)	
Dewormed:				
Yes	91 (24.5)	51 (56.0)	1.00	0.014
Never	280 (75.5)	196 (70.0)	1.83 (1.12-2.98)	
Dog feces management:				
Yes	59 (15.9)	32 (54.2)	1.00	0.029
No	312 (84.1)	215 (68.9)	1.87 (1.06-3.29)	

CI, confidence interval.

### 3.2 Prevalence and molecular characterization of hookworm species

Of the 371 dogs examined, 247/371 (66.6%, 95% CI: 61.5-71.3) were positive for hookworm eggs by fecal flotation. The PCR-RFLP was able to successfully amplify hookworm DNA in 232 samples (93.9%). The remaining 15 samples were positive by flotation but yielded negative results by PCR. Among the 232 positive samples, 191 samples (82.3%) were identified as single hookworm infections with *A. ceylanicum*, while 34 samples (14.7%) were single infections with *A. caninum*, and 7 samples (3.0%) harbored mixed infections with both *A. ceylanicum* and *A. caninum*. No amplification products were obtained for either *A. braziliense* or *U. stenocephala*.

Among the 191 samples identified as *A. ceylanicum* by PCR-RFLP, 188 (98.4%) were successfully amplified for the mitochondrial *cox1* gene. From the successfully amplified samples, 15 were randomly selected for DNA sequencing and subsequent phylogenetic analysis.

### 3.3 Associated factors for hookworm infection in dogs

All explanatory variables included in the analysis were available for all 371 dogs. Univariate analysis revealed that local breed (crude OR [cOR] = 2.21, 95% CI: 1.16-4.18, *p* = 0.013), lack of deworming (cOR = 1.83, 95% CI: 1.12-2.98, *p* = 0.014), and absence of dog fecal management (cOR = 1.87, 95% CI: 1.06-3.29, *p* = 0.029) were significantly associated with hookworm infection in dogs (**Table 1**). In the multivariable analysis, lack of deworming was associated with higher odds of hookworm infection compared with dogs that had been dewormed (adjusted OR [aOR] = 1.77, 95% CI: 1.08-2.89, *p* = 0.022). Similarly, dogs in households without proper fecal management had higher odds of infection (aOR = 1.79, 95% CI: 1.01-3.17, *p* = 0.046) compared to those with proper fecal management. The logistic regression models showed adequate goodness-of-fit based on the Pearson goodness-of-fit test (χ^2^ = 371.07, df = 367, *p* = 0.431). No evidence of multicollinearity was observed among the explanatory variables. One potentially influential observation (observation 122) was identified based on Cook’s distance. Excluding this observation resulted in minimal changes in the estimated associations and did not change their statistical significance.

### 3.4 Phylogenetic analysis of *cox1* in *A. ceylanicum*

Phylogenetic analysis based on the *cox1* gene showed that all sequences obtained in this study clustered within the *A. ceylanicum* clade and were clearly separated from *A. duodenale* and *A. braziliense* (**Figure 1**). The canine isolates from this study did not form a distinct cluster but were interspersed with reference sequences from humans, dogs, and cats reported in several Asian countries. Minor sequence variation was observed among the study isolates. Representative *cox1* sequences generated in this study were submitted to GenBank under accession numbers PZ988225-PZ988239 (**Supplementary Table S1**).

**Figure 1:**
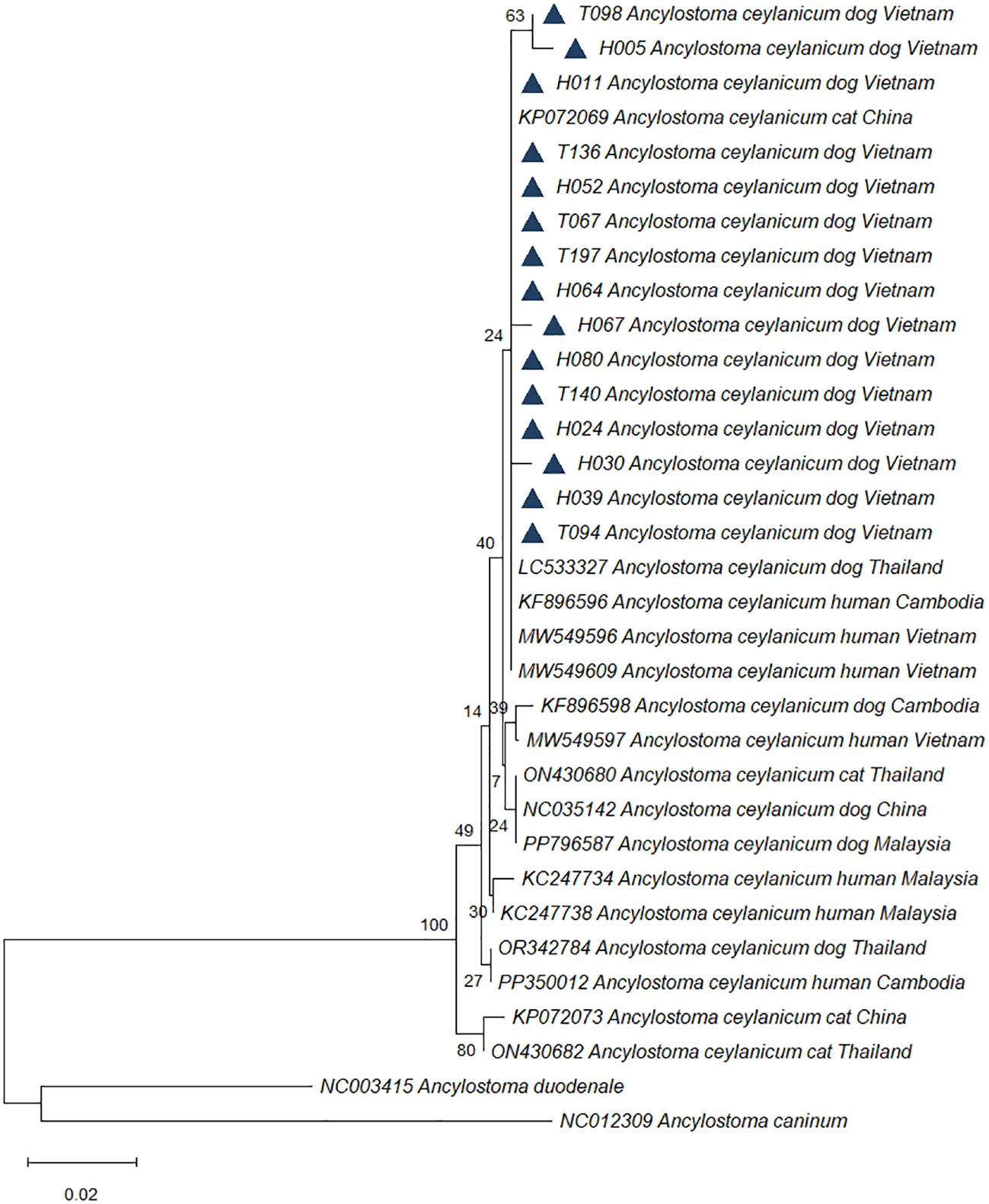
Neighbor-joining phylogenetic tree based on partial *cox1* gene sequences of *A. ceylanicum*. Evolutionary distances were calculated using the Neighbor-Joining model with 1,000 bootstrap replicates. The final alignment comprised 317 nucleotide positions. A phylogenetic tree was performed using MEGA 12 software. Sequences obtained from dogs in the study are indicated in blue triangles.

## 4 Discussion

Canine hookworms are common intestinal parasites of dogs, and some species have zoonotic potential, making them a concern for both veterinary and public health. In the present study, copromicroscopic examination revealed a relatively high prevalence of hookworm infection in dogs. This prevalence was higher than those reported in previous studies conducted in Vietnam (Dinh and Kieu, 2022; Hieu et al., 2016; Na et al., 2025) and in neighboring countries, including Thailand (Kladkempetch et al., 2020), Cambodia (Zendejas-Heredia et al., 2023), and Malaysia (Tahar et al., 2025). In contrast, a higher prevalence was reported in Lao (Conlan et al., 2012). Canine hookworm infections have also been reported in other regions worldwide, with prevalence estimates of 4.5% in Australia, 9.2% in Italy, and 3.2% in Western Europe (Drake et al., 2022; Illiano et al., 2023; Massetti et al., 2022). A systematic review and meta-analysis estimated the pooled prevalence of hookworm infection among domestic dogs in Asia to be 41.0% (Zibaei et al., 2020). The high prevalence observed in the present study is likely associated with tropical environmental conditions and suboptimal dog management practices, including free roaming, contact with other animals, and poor fecal management (**Table 1**), which may facilitate ongoing environmental contamination and transmission.

Molecular analysis in the present study identified two canine hookworm species, including *A. ceylanicum* and *A. caninum*, in dog fecal samples, with *A. ceylanicum* being the dominant species. These finding is consistent with previous surveys conducted in Vietnam, including studies from Phu Tho and Dak Lak provinces (Ng-Nguyen et al., 2015; Nguyen et al., 2022). In contrast, a study from Long An province detected *A. braziliense* in addition to *A. ceylanicum* and *A. caninum* in dogs, with *A. caninum* identified as the predominant species (Chuc et al., 2016). Similar patterns have been reported in several Asian countries, where *A. ceylanicum* and *A. caninum* are commonly identified in dogs, although the predominant species varies by geographic area, host population, and diagnostic approach (Fu et al., 2019; Mahdy et al., 2012; Sayasone et al., 2026; Tahar et al., 2025). The predominance of *A. ceylanicum* in the present study is epidemiologically important because this species is capable of establishing patent infections in humans and is increasingly recognized as a neglected zoonotic hookworm in Asia (Haydon and Bearup, 1963; Traub et al., 2021). Therefore, the detection of *A. ceylanicum* in dogs from this rural community supports the possible role of dogs as reservoirs for zoonotic hookworm transmission, particularly in settings where free-roaming dogs, poor fecal management, and close human-dog contact are common.

Phylogenetic analysis based on *cox1* sequences showed that all *A. ceylanicum* isolates from dogs in the present study clustered within the zoonotic haplotype, together with previously reported sequences from humans in Vietnam and from humans, dogs, and cats in other Asian countries (**Figure 1**). However, the limited availability of dog-derived *A. ceylanicum* reference sequences from Vietnam restricted direct comparison with canine haplotypes circulating locally. This clustering pattern is consistent with the potential involvement of dogs in the transmission of *A. ceylanicum* and suggests possible zoonotic exposure in Vietnam. The public health relevance of these findings is further supported by reports that *A. ceylanicum* is the second most prevalent hookworm species in human populations in Vietnam (Bui et al., 2021; Dyer et al., 2022). A similar pattern has been observed in other Asian countries, where the occurrence of *A. ceylanicum* in dogs overlaps with that in humans (Inpankaew et al., 2014b; Sayasone et al., 2026; Tenorio et al., 2024). Because *A. ceylanicum* is capable of infecting humans and has been reported in both humans and animals, its predominance among the hookworm species identified in this study is of particular public health concern in Vietnam. The phylogenetic findings support the need for integrated surveillance of human and animal populations to better characterize the distribution and potential transmission pathways of *A. ceylanicum* in endemic areas. Such integrated surveillance could facilitate identification of hookworm species circulating in both humans and animals, allowing comparison of their distribution and genetic characteristics across hosts. This approach could help determine whether the zoonotic species, such as *A. ceylanicum*, and genetically related parasite populations are present in humans and animals within the same geographic areas, providing evidence of potential epidemiological links and shared exposure pathways that could inform targeted control strategies.

Multivariable analysis showed that dogs without a history of deworming had higher odds of hookworm infection than those that had received anthelmintic treatment. This finding is consistent with previous studies (Na et al., 2025) and highlights the importance of regular deworming in reducing parasite transmission. In the study area, dogs were commonly allowed to roam freely; therefore, untreated infected dogs may serve as continuous sources of environmental contamination. Poor management of dog feces was also significantly associated with increased odds of hookworm infection. Improper disposal of dog feces facilitates the accumulation of hookworm eggs and larvae in the environment, thereby increasing the likelihood of exposure for other dogs through contact with contaminated soil. These factors are commonly observed in rural communities where dogs have close contact with humans and the environment. Based on these findings, regular deworming of dogs, proper disposal of dog feces, and improved dog management should be promoted to reduce transmission. From a One Health perspective, these measures may help reduce hookworm transmission in dogs and lower the potential risk of zoonotic exposure in humans.

Of the 247 samples positive by fecal flotation, 232 (93.9%) were successfully amplified by PCR, whereas 15 samples failed to yield PCR amplification. Several factors may explain this discrepancy. The most plausible explanation is the morphological resemblance between hookworm eggs and those of *Trichostrongylus* or eggs of plant-parasitic nematodes such as *Meloidogyne* (**Figure 2**). Although eggs of these species are typically larger in size, overlap in size and morphology may still lead to misidentification during microscopic examination (Bradbury and Speare, 2015; Yong et al., 2007). In Vietnam, *Meloidogyne* eggs have been documented in soil samples (Hoang et al., 2021; Trinh et al., 2009), and *Trichostrongylus* is reported in cattle from this province (Dinh et al., 2023). Given that dogs in the study area are typically free-roaming, coffee plant is the main crop, and cattle husbandry is common, environmental contamination with morphologically similar eggs may occur. Another possible explanation that cannot be excluded is low-intensity infections, in which uneven egg distribution in fecal material, combined with the smaller fecal aliquot used for conventional PCR compared with flotation, may result in PCR-negative but flotation-positive samples. Further investigations are therefore needed to resolve these uncertainties.

**Figure 2:**
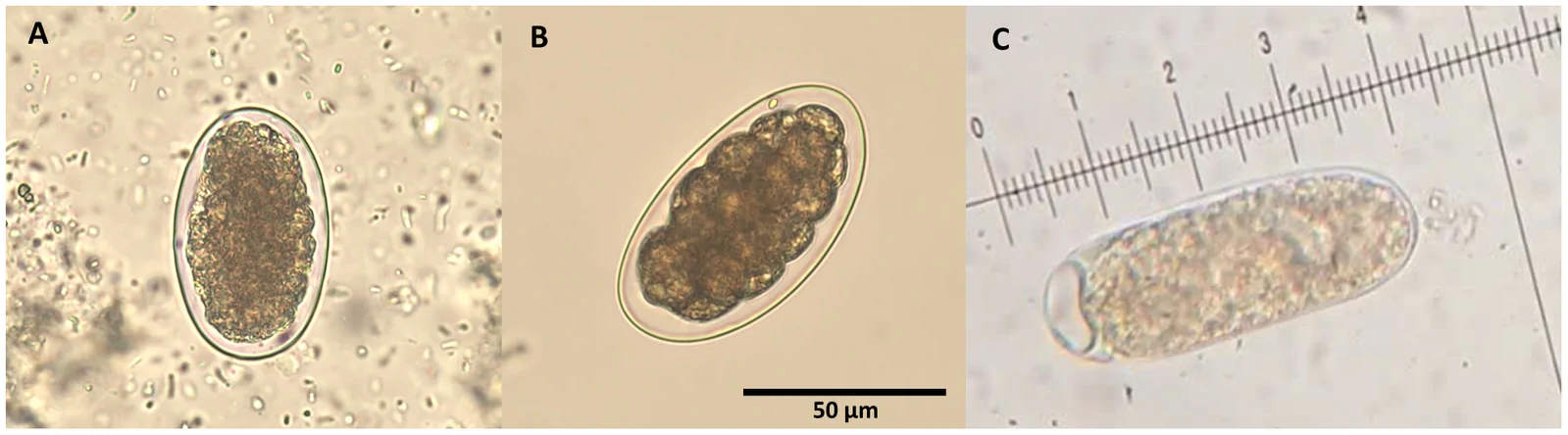
Representative nematode eggs for comparison of morphological features (400× magnification). **(A)** Hookworm egg; **(B)** *Trichostrongylus* egg; **(C)** *Meloidogyne* egg.

The study has several limitations. First, the study was conducted in only two communes in Krong Nang district, Dak Lak province, which may limit the representativeness of the results and their generalizablility to other geographic areas. Second, although dog-owning households were randomly selected from a sampling list, the list may not have captured all eligible households; therefore, selection bias cannot be completely excluded. Third, the study focused on dog samples and did not include human or environmental sampling, precluding direct assessment of zoonotic exposure and environmental contamination with hookworms. Future studies involving broader geographic coverage and integrated sampling of dogs, humans, and the environment would provide a more comprehensive assessment of hookworm populations and provide a more representative understanding of their distribution.

## 5 Conclusion

This study indicated a high prevalence of hookworm infection in dogs in Krong Nang district, Dak Lak province, Vietnam. Two hookworm species, *A. ceylanicum* and *A. caninum*, were identified, with *A. ceylanicum* being the predominant species. Given the zoonotic potential of *A. ceylanicum*, these findings highlight the potential risk of zoonotic transmission in rural endemic settings. Improved dog management, particularly regular deworming and proper disposal of dog feces, may help reduce environmental contamination and limit opportunities for transmission to humans and animals.

## Data Availability

The datasets presented in this study can be found in online repositories. The names of the repository/repositories and accession number(s) can be found in the article/**Supplementary Material**.
