# Supplementary material for: Canine hookworm infections in Vietnam: prevalence, risk factors, and detection of zoonotic Ancylostoma ceylanicum
Source: Front Cell Infect Microbiol. 2026 Oct 28;16:1926338. doi: 10.3389/fcimb.2026.1926338 (PMC13751462; doi:10.3389/fcimb.2026.1926338)
Supplement: Supplementary file 1 [file Table1.docx]

| **GenBank accession number** | **Isolate ID** |
| --- | --- |
| PZ988225 | H005 *Ancylostoma ceylanicum* dog Vietnam |
| PZ988226 | H011 *Ancylostoma ceylanicum* dog Vietnam |
| PZ988227 | H024 *Ancylostoma ceylanicum* dog Vietnam |
| PZ988228 | H030 *Ancylostoma ceylanicum* dog Vietnam |
| PZ988229 | H039 *Ancylostoma ceylanicum* dog Vietnam |
| PZ988230 | H052 *Ancylostoma ceylanicum* dog Vietnam |
| PZ988231 | H064 *Ancylostoma ceylanicum* dog Vietnam |
| PZ988232 | H067 *Ancylostoma ceylanicum* dog Vietnam |
| PZ988233 | H080 *Ancylostoma ceylanicum* dog Vietnam |
| PZ988234 | T067 *Ancylostoma ceylanicum* dog Vietnam |
| PZ988235 | T094 *Ancylostoma ceylanicum* dog Vietnam |
| PZ988236 | T098 *Ancylostoma ceylanicum* dog Vietnam |
| PZ988237 | T136 *Ancylostoma ceylanicum* dog Vietnam |
| PZ988238 | T140 *Ancylostoma ceylanicum* dog Vietnam |
| PZ988239 | T197 *Ancylostoma ceylanicum* dog Vietnam |
